# Pseudogenes and host specialization in the emergent bacterial plant pathogen *Xylella fastidiosa*

**DOI:** 10.1128/aem.02070-24

**Published:** 2025-04-10

**Authors:** Navdeep Kaur, Neha Potnis, Leonardo De La Fuente

**Affiliations:** 1 Department of Entomology and Plant Pathology, Auburn University 1383, Auburn, Alabama, USA; University of Tennessee at Knoxville, Knoxville, Tennessee, USA

**Keywords:** Pseudofinder, intergenic, weeds, host range, host generalists, host specialist

## Abstract

Pseudogenes are regarded as “junk” DNA, representing vestigial functions no longer needed for fitness. Accordingly, a higher number of pseudogenes in a bacterial human pathogen was previously hypothesized to be a hallmark of host specialists. In this study, we tested this hypothesis on the emergent bacterial plant pathogen *Xylella fastidiosa* (*Xf*) to link pseudogene makeup and host specificity. *Xf* is an ideal subject for these studies by being a xylem-limited pathogen that underwent extensive genome reduction. Using natural host range data of 151 strains and Pseudofinder analysis on *Xf* whole genome sequences, we observed that *Xf* subsp. *sandyi* had the highest pseudogene content, followed by subsp. *morus*, while subsp. *pauca*, *fastidiosa*, and *multiplex* had the lowest. The first two subspecies are known to have a limited host range compared to the others, aligning with the hypothesis of a greater number of pseudogenes corresponding to narrower host range. Weed isolates are presumably host specialists because they had the highest pseudogene content. Using a thorough pseudogene map across genomes and empirical pathogenicity data on blueberries, we screened for genes potentially involved in blueberry specialization. Targets were identified by selecting sequences pseudogenized (i) in strains infecting hosts different from blueberry and (ii) only in blueberry asymptomatic strains. Six sequences were identified with a potential role in blueberry infection, including one that was common between the two criteria. Here, we generated hypotheses on host range and specificity of *Xf* strains that need to be tested experimentally to help understand this devastating plant pathogen.

**IMPORTANCE:**

*Xylella fastidiosa* is a highly destructive plant pathogen that infects hundreds of landscape and agriculturally important plant species mainly in Europe and the Americas. Nevertheless, the host range of specific genotypes and underlying mechanisms of host specificity remain unclear. These are important aspects to determine the potential risk of infection in specific areas depending on the genetic makeup of the pathogen population and hosts present. This study offers valuable insights into the role of pseudogenization in the genomes of different *X. fastidiosa* strains, linking it to host specialization. Despite the limited information available for the host range of different strains of this pathogen, this research proposes a relationship between the abundance of pseudogenes and host specificity. These findings are essential for predicting potential host shifts by this pathogen, aiding in the development of strategies to prevent its spread. Additionally, the identification of candidate genes putatively important for symptom development in blueberries offers targets for prevention and control efforts.

## INTRODUCTION

The Lysobacteraceae (syn: Xanthomonadaceae) bacterium *Xylella fastidiosa* poses a serious threat to agricultural crops, ecosystems, and ornamental landscapes worldwide (1–3). This bacterium is responsible for multiple plant diseases including Pierce’s disease (PD) of grapevine (4), bacterial leaf scorch of blueberries (5), citrus variegated chlorosis (CVC) (6), and olive quick decline syndrome (OQDS) (7). *X. fastidiosa* was historically restricted to the Americas, but it has recently spread to the European and Asian regions, possibly due to the importation of infected plant material (8). The majority of *X. fastidiosa* strains are currently divided into three main subspecies: *X. fastidiosa* subsp. *fastidiosa, pauca,* and *multiplex*, which are thought to have originated in Central America, South America, and North America, respectively (2). The existence of two additional North American-native subspecies, *X. fastidiosa* subsp. *sandyi* and *morus*, has been suggested (9). Currently known to infect 712 plant species, *X. fastidiosa* infections can be symptomatic or asymptomatic in different host plants (10). This bacterium colonizes exclusively the xylem vessels of plant hosts and mouth parts of insect vectors (11), being obligately transmitted by Hemipteran insects that feed on xylem sap (1).

Due to the long time needed and technical difficulties of performing experimental virulence assessments for *X. fastidiosa* (12), just a few studies (13–15) have tested the host range of genetically distinct *X. fastidiosa* strains. Results indicate that most *X. fastidiosa* strains tested show host specificity, but the molecular bases of specificity are unknown. A few *X. fastidiosa* strains have been tested for reciprocal infection, and results indicate that some grape strains cannot infect oleander and vice versa (16). Similar results were found with peach and grape strains (17). Nevertheless, some strains isolated from grapes were able to infect almonds (18) and blueberries (14), while some almond strains can also infect blueberries (19). These limited studies highlight the need to understand the host range of specific strains to anticipate possible host jumps.

In many bacterial–host interactions, the secretion of effectors by type three secretion system (T3SS) and its interactions with the plant host have been demonstrated as the basis for host specificity (20). However, *X. fastidiosa* lacks T3SS (21); therefore, it is not possible to predict host specificity based solely on genome sequence until we understand the basis of these interactions. A loose association of *X. fastidiosa* subspecies classification and host specificity has been suggested (22), yet some strains can infect multiple hosts, and a particular host can be infected by multiple subspecies (23). However, the mechanisms by which different strains of *X. fastidiosa* infect or colonize specific plant hosts and no other hosts are unclear.

Pseudogenes, or disabled copies of genes, are frequently found in the genome of organisms that have recently stopped using them, and they are characterized by premature stop codons and/or frameshifts (24). Pseudogenes are a common feature and are found even in the smallest of bacterial genomes (25). As an organism adjusts to a new environmental niche, it is common for pseudogene accumulation to indicate recent adaptation to a new host (26). Genomic comparisons among animal pathogen *Salmonella enterica* serovars provide clear illustrations of this hypothesis. The number of pseudogenes in host-specialist serovars of *S. enterica* was found to be higher than in host-generalist serovars. Therefore, pseudogenes were regarded as distinctive features of host specialists (27). In a preliminary assessment of pseudogenes in bacterial plant pathogens, we identified that pseudogenes were more common in xylem-limited bacteria than in facultative xylem colonists—those that inhabit other niches besides the xylem (28). This finding suggests a genome reduction and niche specialization in xylem-limited pathogens. Interestingly, compared to other xylem-limited bacteria, *X. fastidiosa* had a lower number of pseudogenes, which is possibly an indication of its ancient association with host plants (29).

In this study, we found a relationship between the percentage of pseudogenes and the reported natural host range among *X. fastidiosa* strains at the subspecies level. This indicates that the number of pseudogenes can be indicative of the generalist or specialist potential of *X. fastidiosa* strains. Recently, various genomic studies on *X. fastidiosa* used phylogenetic approaches to identify genes possibly associated with host specificity (30–32). We followed a different approach and focused on identifying genes that have been pseudogenized in strains of *X. fastidiosa* not infecting certain hosts but are still functional in strains infecting that host, as an indication of host specificity. For this, we focused on strains known to cause symptoms or asymptomatic infections in blueberries, and we identified a few sequences that could have a role in symptoms development in this host. We expect that our study will generate further research into using pseudogene information to predict the host range on *X. fastidiosa* and determine the basis of host specificity.

## MATERIALS AND METHODS

### Genomes used in this study

Whole genome assemblies of each strain used in this study were either downloaded from GenBank (33) or directly obtained from the authors (30) (Table S1). All these genomes were first annotated by Prokka (34). The quality of genomes was assessed using CheckM (35). A total of 151 genomes from five different subspecies, i.e., *X. fastidiosa* subsp. *fastidiosa* (*n* = 67)*,* subsp. *multiplex* (*n* = 53)*,* subsp. *pauca* (*n* = 22)*,* subsp. *morus* (*n* = 6)*,* and subsp. *sandyi* (*n* = 3), were used in this study, which included diversity with respect to the country of isolation and host plant species from which they were originally isolated. These strains were isolated in the USA (*n* = 74), Spain (*n* = 37), Brazil (*n* = 12), Italy (*n* = 12), Costa Rica (*n* = 6), France (*n* = 5), Taiwan (*n* = 4), and Mexico (*n* = 1). Host plants from which these strains were isolated include grapes (*n* = 52), almond (*n* = 24), olive (*n* = 12), coffee (*n* = 9), weed/wild (*n* = 8), citrus (*n* = 7), mulberry (*n* = 6), blueberry (*n* = 5), plum (*n* = 5), buckthorn (*n* = 5), and a category named “others” (*n* = 18) that compiles strains from which few genomes were available per host. The “others” category includes strains isolated from host species such as oleander (*n* = 3), Spanish broom (*n* = 4), oak (*n* = 1), hibiscus (*n* = 1), cherry (*n* = 1), curry plant (*n* = 2), western redbud (*n* = 1), myrtle leaf milkwort (*n* = 3), pecan (*n* = 1), and plane tree (*n* = 1). Strains isolated from periwinkle (*n* = 1), common ragweed (*n* = 2), elderberry (*n* = 1), lupine (*n* = 1), sumpweed (*n* = 1), sunflower (*n* = 1), and fig (*n* = 1) were assigned to weed/wild category (36). For this study, the host of isolation was considered the only host of that particular strain, as there is a lack of information on the host range of most *X. fastidiosa* strains.

### Pseudogene analysis

The open-source software Pseudofinder (37) was used for generalized pseudogene prediction in genomes of *X. fastidiosa*. A reference-based approach is used by this tool to detect a wide variety of pseudogene-inducing substitutions and gene structures, where customizable options are available to users for defining pseudogenes. In this study, non-redundant protein databases were used as a reference to interrogate pseudogenes at detailed gene-to-gene level (37). Pseudofinder’s core pipeline called “Annotate” was used to detect candidate pseudogenes. *X. fastidiosa* genomes in GenBank format (NCBI compliant, with both gene and coding sequence [CDS] features) and protein sequence database were used as input, with the default parameters (37). The output file gave the total number of pseudogenes (Table S1) obtained from the blast hits with non-redundant protein database. The percentage of candidate pseudogenes was first calculated by dividing the total number of pseudogenes by the total number of genes. The total number of genes was calculated as the total number of intact genes plus candidate pseudogenes. The total number of pseudogene sequences was categorized by this pipeline into (i) “Truncated/Short” by comparing CDS lengths to the average length of their blast hits, flagging CDS that was significantly shorter as potential pseudogenes; (ii) “Long/Run-on” where CDS exceeded the average length of their blast hits, often indicative of stop codon loss; (iii) “Fragmented” by assessing neighboring CDS to identify instances where multiple adjacent CDS shared numerous common blast hits, suggesting they were fragments of a single ancestral gene; and (iv) “Intergenic” by extracting nucleotide sequences between annotated features and conducting BLASTX analysis across all six reading frames (37).

Average lengths of different types of pseudogenes, i.e., short, long, fragmented, and intergenic pseudogenes, were calculated only for the model strain *X. fastidiosa* subsp. *fastidiosa* Temecula1 to understand the process of pseudogenization. The list of pseudogenes generated with Pseudofinder for strain Temecula1 was also cross-examined by comparison with our previously generated whole transcriptome data *in vitro* for this strain using RNA-Seq (38). For that study, strain Temecula1 was grown in Erlenmeyer flasks *in vitro* for 3 days using two different media: PD2 and PD2 supplemented with calcium. Biofilm cells were collected every 24 hours from each flask, and the whole transcriptome was analyzed for both media and three time points (38). For the current study, we first compiled the gene sequences transcribed by cells growing in biofilms only under PD2 media conditions at three different time points, i.e., 24, 48, and 72 hours (38). We used this compiled list of genes as described in a previous study (38), without additional modification. Then, Conditional Reciprocal Best BLAST (CRB-BLAST) (39) was performed using pseudogene sequences of Temecula1 as a query against the list of genes transcribed taken from the previous study (38). We hypothesized that transcribed genes will not be found in the pseudogenes list. Pseudogene sequences having a sequence identity of ≥90% with the transcribed sequences were selected.

### Phylogenetic analysis

Using Roary, the core genome alignment of all the strains was obtained to create a phylogenetic tree (40). A generalized time-reversible model was used to create a maximum likelihood phylogeny using RAxML V8.2.11. One thousand bootstrap replicates were utilized to assess node support. The Interactive Tree of Life was employed for tree visualization (41). Host species and country of isolation were also included in the phylogenetic tree. To better illustrate the variation in the proportion of pseudogenes among the strains, bars displaying the percentage of pseudogenes (rounded to whole number) corresponding to each strain were added to the phylogenetic tree.

### Comparison of pseudogenes in different host groups against blueberry strains

Pseudogenes were compared among strains from different hosts with the goal of identifying intact genes in blueberry strains that were pseudogenized in others, as an indication of a possible role in blueberry specificity. The blueberry group was used as a central point to compare to all other host groups mentioned above, i.e., grapes, almond, olive, citrus, coffee, mulberry, plum, and buckthorn (except “weed/wild” and “others”). Blueberry is an economically important fruit crop in the United States, and since the increased production in Southeastern states of the United States over the last decades, it has been affected by *X. fastidiosa* (42, 43). Pseudofinder distinguishes between putative pseudogene calls and intact genes, so shared intact gene sequences in all five blueberry strains were identified, and those shared intact sequences were combined into one file to use as a database. These sequences were considered *a priori* as candidates for blueberry specificity. Then, pseudogene sequences of each strain from non-blueberry hosts were compared against the list of shared intact sequences among all blueberry strains using the CRB-BLAST, and pseudogene sequences showing ≥90% sequencing similarity were selected for further analysis. After obtaining the pseudogene sequences of each strain (that were intact in all blueberry strains), a database was created with those pseudogene sequences, and shared pseudogene sequences were identified among the strains belonging to the same host group. Then, shared pseudogene sequences were determined between different host groups, with each and every possible pair: mulberry–coffee, mulberry–citrus, mulberry–almond, mulberry–olive, citrus–coffee, citrus–olive, coffee–olive, almond–coffee, almond–olive, citrus–almond, coffee–plum, coffee–buckthorn, mulberry–buckthorn, mulberry–plum, citrus–buckthorn, citrus–plum, olive–buckthorn, olive–plum, almond–buckthorn, almond–plum, and plum–buckthorn. This was our first criterion to identify the pseudogene candidates that overlap between different host groups, which were considered as not needed for *X. fastidiosa* infection of the host groups considered, but their intact gene counterparts, if present in the test group blueberry, may be involved in host specificity. To find the shared pseudogene sequences between different host groups, CRB-BLAST was used with a sequence similarity of ≥90%.

### Comparison between symptomatic and asymptomatic strains on blueberry

To identify additional genes in blueberry strains with a potential host specificity role, we used an additional criterion as the one described above. In our previous greenhouse studies, we have tested a few strains belonging to different subspecies for virulence on blueberry plants. Among those, *X. fastidiosa* subsp. *pauca* De Donno, *X. fastidiosa* subsp. *pauca* XYL1961, *X. fastidiosa* subsp. *multiplex* XYL466/19, *X. fastidiosa* subsp. *multiplex* XF3348 (19), and *X. fastidiosa* subsp. *fastidiosa* EB92.1 (14) did not cause any symptoms, so these were considered as asymptomatic strains here. We compared the pseudogene sequences of these five asymptomatic strains with intact gene sequences of symptomatic *X. fastidiosa* subsp. *multiplex* strains AlmaEm3, BB08-1, LA-Y3C, BB01, and BBI64, and the resulting sequences were assessed on NCBI to obtain their annotation by using the reference genome of AlmaEm3.

### Statistical analysis

The statistical analysis for the percentage of total pseudogenes was performed using the *R* version 4.2.2 (44) and *RStudio* (45). Different packages such as ggpubr and ggplot2 were used for statistical analysis and creating plots. First, the normality test was performed using Shapiro’s normality test. All data had a non-parametric distribution; therefore, the Kruskal-Wallis test (46) was used. The significance level of *P* < 0.05 was chosen as a threshold for significant differences in the treatment groups. Pairwise Wilcoxon test (47) was used to calculate pairwise comparisons between group levels. For the statistical analysis of different types of pseudogenes, the JMP Pro version 17 (48) software was used.

## RESULTS

### Variation in pseudogene content among different strains of *X. fastidiosa*

**Fig 1:**
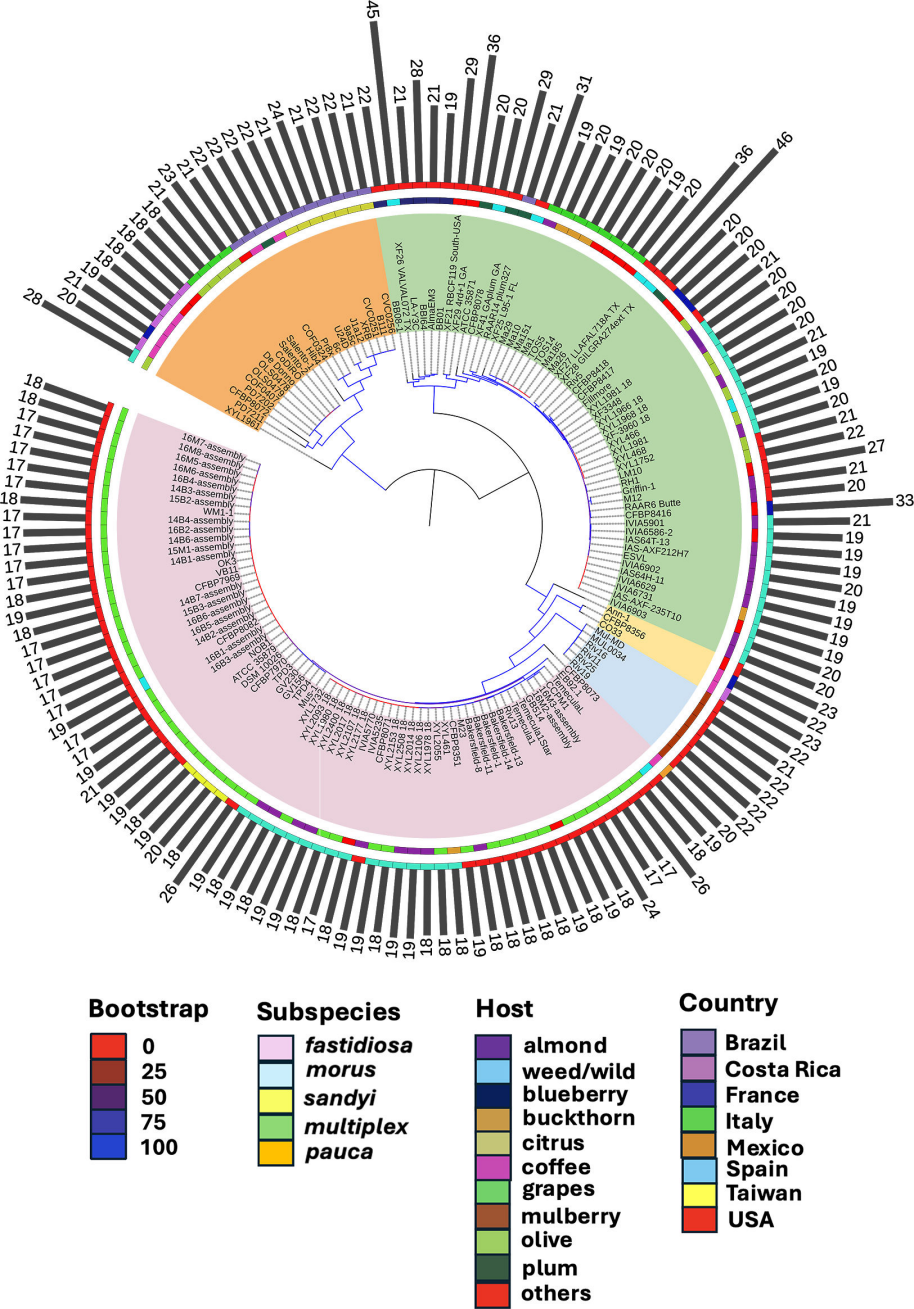
Maximum likelihood phylogeny of core genome alignment of 151 strains of *X. fastidiosa*. Each strain was classified into subspecies shown in different colors in the most inner ring. The second ring from the center shows the plant host of isolation of each strain, while the next ring indicates the countries of isolation. All this information is color coded according to the keys shown in the figure. The percentage of pseudogenes (rounded to whole number) is represented in the outermost ring as bars in black color. Dashed lines were used to connect the tips of the phylogeny to the strain names. The range of bootstrap values is shown with color-coded branches on the tree (see key above), which were obtained after 1,000 replicates. Detailed information can be found in Table S1.

The percentage of pseudogenes in the genomes of *X. fastidiosa* strains ranged from 17% to 46%. For *X. fastidiosa* subsp. *fastidiosa*, pseudogene numbers in most strains were very close, ranging from 17% to 19%. Only a few strains had slightly higher percentages: GV156 and CFBP8073 (20%), ATCC_35879 (21%), GB514 (24%), and CCPM1 and Mus-1 (26%). For *X. fastidiosa* subsp. *multiplex*, most of the strains had pseudogenes between 19% and 21%. The strains that had higher percentages were RH1 and BB08-1 (22%), Griffin-1 (27%), BBI64 (28%), XF41_GAplum_GA and XF21_RBCF119_South_USA (29%), XF25_L-95–1_FL (31%), CFBP8416 (33%), and XF27_LLAFAL718A_TX and XF29_4rd+1_GA (36%). Notably, *X. fastidiosa* subsp. *multiplex* strain XF28_GILGRA274ext_TX, isolated from sunflower (*Helianthus* sp.), showed the highest (46%) percentage of pseudogenes among all strains tested (Fig. 1). Another strain with an exceptionally high number of pseudogenes was *X. fastidiosa* subsp. *multiplex* strain XF26_VALVAL072_TX (isolated from Ragweed, *Palmer amaranthus*) that contains 45% pseudogenes. Strains classified in *X. fastidiosa* subsp. *pauca*, *X. fastidiosa* subsp. *sandyi*, and *X. fastidiosa* subsp. *morus* had pseudogene ranges of 18%–24%, 22%–23%, and 21%–22%, respectively (Fig. 1).

### Model strain *X. fastidiosa* subsp. *fastidiosa* Temecula1

**Fig 2:**
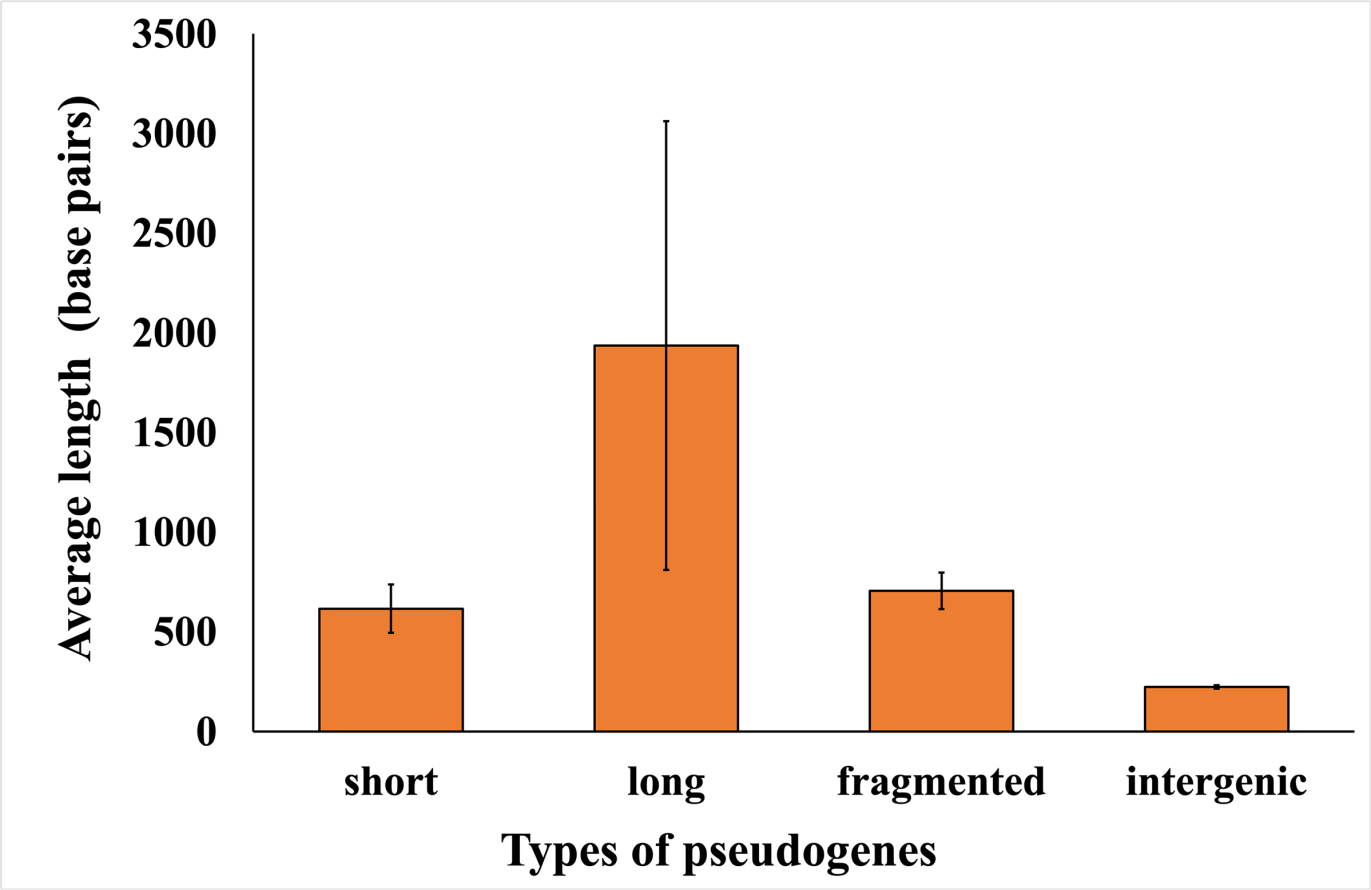
Average length of nucleotides in different categories of pseudogenes in *X. fastidiosa* subsp. *fastidiosa* strain Temecula1 as differentiated by Pseudofinder. Values indicate the average length of pseudogenes categorized by type in the model strain *X. fastidiosa* subsp. *fastidiosa* Temecula1. Error bars represent standard errors.

We focused on *X. fastidiosa* subsp. *fastidiosa* strain Temecula1 as a model strain that has been used for multiple studies of Pierce’s disease *in vitro* and *in planta*, and for which whole transcriptome data were available. First, the average lengths of distinct pseudogene types as identified by Pseudofinder were calculated. Pseudogenes were classified into four different types by Pseudofinder—short, long, fragmented, and intergenic. Among these pseudogene types, the long pseudogenes exhibited an average length of 1,936 bp, followed by the fragmented pseudogenes at 706 bp, and the short pseudogenes at 616 bp. Finally, pseudogenes categorized as intergenic displayed the shortest average length at 224 bp (Fig. 2).

In a previous study, our group used RNA-Seq to describe the whole transcriptome of *X. fastidiosa* subsp. *fastidiosa* strain Temecula1 grown under different *in vitro* conditions (38). Using data from the growth on PD2 media condition at three different timings (24, 48, and 72 hours), we confirmed that ~98% of the 441 pseudogene sequences classified as pseudogenes in this strain by Pseudofinder were not transcribed. Nevertheless, nine (~2%) of the sequences classified as pseudogenes were actively transcribed (Table 1). The level of expression of these nine sequences was similar to the expression of other genes at the three different time points mentioned above (38). Five of the nine sequences belonged to the “hypothetical protein” category, whereas *yggB* was categorized as a secondary transporter, *cysD* has a function in energy metabolism, and two other sequences were annotated as phage-related proteins (38). From all these sequences, *cysD* was a fragmented pseudogene, whereas the rest of the sequences were intergenic pseudogenes as classified by Pseudofinder.

**TABLE 1:** Sequences identified as pseudogenes but transcribed in *X. fastidiosa* subsp. *fastidiosa* strain Temecula1

Pseudogene sequence^*a*^	Annotation^*b*^	Locus tag^*b*^
<Seq(i) GAGGATGTGGATCCAGTCGATCCGTGTCCAGTTGAATCTGCCGTGGAA CGTCGTCACTGTTGCAGGCGATACGATGTTTGATAGTAATTTAGTCCAGTCCA	*yggB*	PD0523
<Seq(ii) ATGTTTTCGGTTGGCTACGCTGTCCGCCGATGTGTACATCACTCCAAT TCTCTAGAGTTGCTACCTCTGATGGATTCATCCTCTTTCTCTTTTTCTC ATCTTGATCGGCTTGAGGCTGAAAGTATCTACATCTTGCGTGAGGTCG TGGCGGAATTCCGTAATCCGGTGTTGCTTTACTCTGTGGGTAAGGATA GTTCAGTACTGTTGCACCTGTTACTTAAGGCGTTTGCGCCGGCACCG CCACCGATTCCGCTACTGCATGTCGATACGCGTTGGAAGTTTCGCGA GATGATTGCCTTTCGTGATCGCCGTGTTGCTGAGACGGGGGTACAGT TGCGTGTCCACATTAATCTTGAGGGTGTGGCGCAGGAGATTAATCCG ATCACTCACGGTGCCGCTGTGCATACGGACATTATGAAAACGCAGGG GTTGCGGCAGGCGTTGGAGCAGGGGCAGTTTGATGCGGCGATTGGT GGAGCACGCCGCGATGAAGAAAAGTCGCGTGCCAAGGAGCGTGTATT CTCGTTTCGTAACGCCCATCATCGATGGGATCCCAAAAACCAACGTCCT GAATTGTGGAATGTGTACAACGCGCGTATTCATCCTGGAGAGAGCGTG CGGGTATTTCCACTGTCTAATTGGACTGAATTGGATGTGTGGTTGTACA TCTATCGTGAGCAGATTCCGGTGGTGTCGTTATATTTTGCTGCACCGCG CCCAGTGGTTGAGCGCGACGGTATGTTGATTCTGGTTGACGATGAGC GTTTGCCCCTGCATCCGGGCGAAGTGTCGAAGTTGCGTTGGGTGCGCT TCCGTACCTTGGGTTGCTATCCCCTGACTGGGGCGGTTGAGTCTCGTGC TGCGACTTTAGAAGACATCATTGCTGAGATGCTGGTGACTCCCTTTTCG GAGCGTCAGGGGCGGCTGATCGATTATGCCCCTGGTGCCTCAATGGAA AGCAAAAAGATAGAGGGGTATTTCTGA	*cysD*	PD0717
<Seq(iii) ATGGTGCATTTTATTCAGAGTGTCTATTCAAAATATGCTTTGATTCGGT CTTTTTTTTATTTTCCGTTTGCTACGCTTTTTATTAATTTTCTCTTTTCGT GTTTTTTGTTGATGTTGGCGTGTGTTTGTTTGGTTTTATTAGTTCTTGTT CAGCCAACTTCTGGTTTTCAGGATTATTTTTTGCATTCTGTGTCTCATTC TTTGTCTTCACCTGTTGCTTCATTGTGTCTTTACACTTTTGCTTTTTCTCT CTGCAATTGTGTTTTTAGAACCTGTCATCTTCTCTGTTGGGGATTATGGG GGCGTCGTGATTCTTTGGCCTTTAATCGTTC	Hypothetical protein	PD0909
<Seq(iv) CGGAGACGCTTTACGCTTTTGTTGGCGTTGTATCAGCACTTGCCTTAATA CGATGATCGACGCGGTTCCATTGGCTGTGTGCCTTCTTTTACGCAAAACC GTTTTTTGAGATGCGTTAG	Phage-related protein	PD0917
<Seq(v) ATGGTGCATTTTATTCAGAGTGTCTATTCAAAATATGCTTTGATTCGGTCTT TTTTTTATTTTCCGTTTGCTACGCTTTTTATTAATTTTCTCTTTTCGTGTTTTT TGTTGATGTTGGCGTGTGTTTGTTTGGTTTTATTAGTTCTTGTTCAGCCAAC TTCTGGTTTTCAGGATTATTTTTTGCATTCTGTGTCTCATTCTTTGTCTTCAC CTGTTGCTTCATTGTGTCTTTACACTTTTGCTTTTTCTCTCTGCAATTGTGTT TTTAGAACCTGTCATCTTCTCTGTTGGGGATTATGGGGGCGTCGTGATTCTT TGGCCTTTAATCGTTC	Hypothetical protein	PD0922
<Seq(vi) CGGAGACGCTTTACGCTTTTGTTGGCGTTGTATCAGCACTTGCCTTAATACGATGATC GACGCGGTTCCATTGGCTGTGTGCCTTCTTTTACGCAAAACCGTTTTTTGAGATGCGTTAG	Phage-related protein	PD0930
<Seq(vii) AAAGAGAAAATTAATAAAAAGCGTAGCAAACGGAAAATAAAAAAAAGACCGAATCAAA GCATATTTTGAATACACACTCCGAATAAAATGCACCA	Hypothetical protein	PD0940
<Seq(viii) ATCCGTGCTCAATAGCAGATGAAACCATGTGTGCATATTGACATTGGAAAGGTGCACA CATCCTATAAAAACGCAGGGAAGTTCAACCATCCCCAATCCAGAACATACAAATATTGA CCTCAGCATATTCAGACCTGTTCGAGCACACCCTTTTAGCACAGTGATTTCTATTGCTG ATTTGTCTCCCTGATAAGCCTCACAACGTCCATCAATGAATATCTTTCTGGAAATTACAT CGTTCTTTGTCAGAGTCAGACAAACGCTTTCTTGCGGTCGTA	Conserved hypothetical protein	PD1145
<Seq(ix) AAAAACCGACACAGCGCCGACTGTCTATGTGACCCCGATGCAAACGTGCACTATGAA AACAACCCTAACATCACTGTAAAATTAGAACGATACAAACATACCGCCGCTCACAGCT GCAATACGCATTGTGACGCAATCGGATCACTGCAGCAGATCAATGCAGCACACCGTG ATCCCTTTAAAATTAAAAAAATGCAGTACACAACGACAACGATGCTCTATCACCACACA CCGCACATGAGCGAACGACACTCCCACCACTTAAGCAATACCGCAACATTGCCATTG CTAGGAAACAACCGCATTAACACCATTCATCATCCCTTCTCCTTCCACAGTAAGCGGC TTAATGCCACGTCACGACATGATGAAGGGATATCGATTTTTTTCTTTACACATTCCAATT CAGCAAGCAACTCGCACACATCAACATCAGCATACGAACAGAAGCACTCGCACACTG AGGCAAGGCAACTGCAAAAACGCCGCACGCATCGTTCACTAAA	Conserved hypothetical protein	PD1816

^*a*^ Sequences were identified by comparing pseudogenes classified by Pseudofinder with whole transcriptome analysis of *X. fastidiosa* subsp. *fastidiosa* strain Temecula1. These nine pseudogene sequences were the only ones found to be transcribed *in vitro*.

^*b*^ Annotation and locus tag information taken from NCBI.

### Pseudogene percentages are variable among subspecies, host, and country of isolation

**Fig 3:**
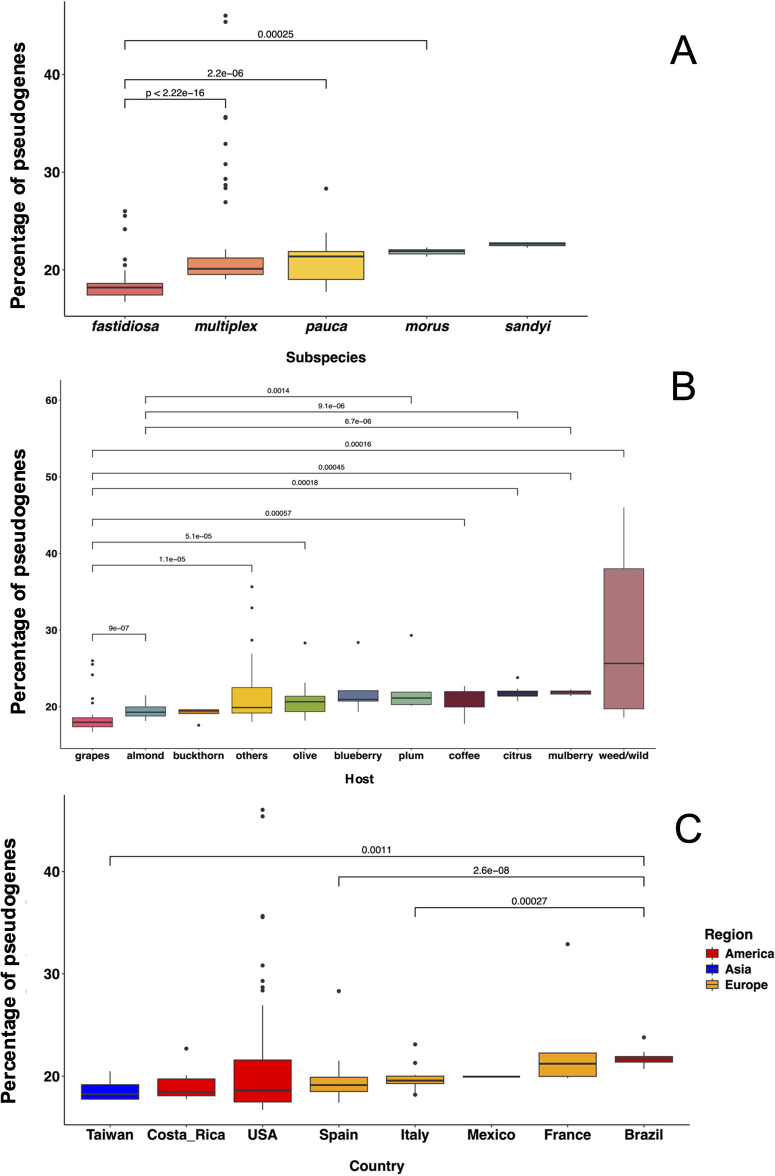
Percentage of pseudogenes in different strains of *X. fastidiosa*. The proportion of total pseudogenes was calculated by dividing the number of pseudogenes by the total number of genes (intact genes and pseudogenes). Strains were grouped based on (**A**) subspecies classification: *fastidiosa* (*n* = 67)*, multiplex* (*n* = 53)*, pauca* (*n* = 22), *morus* (*n* = 6)*,* and *sandyi* (*n* = 3). (**B**) Host of isolation: grapes (*n* = 52), almond (*n* = 24), others (*n* = 18), olive (*n* = 12), coffee (*n* = 9), weed/wild (*n* = 8), citrus (*n* = 7), mulberry (*n* = 6), blueberry (*n* = 5), buckthorn (*n* = 5), and plum (*n* = 5). (**C**) Country of isolation: USA (*n* = 74), Spain (*n* = 37), Brazil (*n* = 12), Italy (*n* = 12), Costa Rica (*n* = 6), France (*n* = 5), Taiwan (*n* = 4), and Mexico (*n* = 1). Different countries are color coded according to geographical regions, i.e., USA (red), Asia (blue), and Europe (orange). Values of *P* < 0.05 were considered as a threshold for statistical significance according to the Kruskal-Wallis test followed by pairwise Wilcoxon test.

Strains classified as *X. fastidiosa* subsp. *sandyi* (22.7%) contained the highest median percentage of pseudogenes, followed by *X. fastidiosa* subsp. *morus,* subsp. *pauca*, and subsp. *multiplex*. On the other hand, *X. fastidiosa* subsp. *fastidiosa* (18.2%) had the lowest median percentage of pseudogenes. Unfortunately, there was a lot of variability in the sample size (number of strains) of each subspecies due to the composition of the strains sequenced and publicly available. The number of outliers was highest in the case of *X. fastidiosa* subsp. *multiplex* where 10 strains were found: XF28_GILGRA274ext_TX, XF26_VALVAL072_TX, XF29_4rd+1_GA, XF27_LLAFAL718A_TX, CFBP8416, XF25_L95-1_FL, XF41_GA_Plum_GA, XF21_RBC119_South-USA, BBI64, and Griffin-1. Among *X. fastidiosa* subsp. *fastidiosa,* five outliers were found: Mus-1, CCPM1, GB514, ATCC_35879, and GV156. Among *X. fastidiosa* subsp. *pauca*, only one outlier strain was identified: XYL1961. Significant differences (*P* < 0.05) were observed in pseudogene percentages between *X. fastidiosa* subsp. *fastidiosa* with *X. fastidiosa* subsp. *multiplex,* subsp. *morus,* and subsp. *pauca* (Fig. 3A).

Among all 11 host categories used here, the weed/wild (25.6%) group had the highest percentage of pseudogenes, while the grapes (18.0%) group had the lowest. Significant differences (*P* < 0.05) were detected between the grape strains and other host groups (citrus, coffee, mulberry, olive, almond, weed/wild, and “others”). Citrus, plum, and mulberry groups had a significantly (*P* < 0.05) higher percentage of pseudogenes as compared to the almond group. Outliers were observed for grapes (five strains): Mus-1, CCPM1, GB514, ATCC_35879, and GV156. The “others” host category had three outlier strains: XF29_4rd+1_GA, CFBP8416, and XF21_RBCF119_South-USA. “Other” hosts had only one outlier strain each: blueberry BB164, buckthorn XYL461, citrus J1a12, olive XYL1961, and plum XF41_GAplum_GA (Fig. 3B).

To determine whether the geographical origin of isolates has any relationship with pseudogene content in different strains, we examined the data based on country of origin. Strains isolated from Brazil (21.7%) showed the significantly highest percentage of pseudogenes as compared to Italy, Spain, and Taiwan. However, no significant differences (*P* < 0.05) were observed between Brazil and Costa Rica, France, Mexico, and USA groups. The sample size was variable for each category, and unfortunately, only one strain from Mexico was available. The USA strains showed a higher range in terms of pseudogene proportion as compared to other location groups. This group included the highest number of outliers (seven), and some strains showed extreme values ~45%–46% of pseudogenized genome (see above, *X. fastidiosa* subsp. *multiplex*) (Fig. 3C).

### Types of pseudogenes

At all three classification levels considered, i.e., subspecies, host of isolation, and country of isolation, the proportion of “intergenic” pseudogenes was found to be the highest among all four different types of pseudogenes. Among all five subspecies, *X. fastidiosa* subsp. *fastidiosa* (92%) had the highest proportion of intergenic pseudogenes. *X. fastidiosa* subsp. *sandyi* (15%) had the highest number of “short” pseudogenes, whereas “long” pseudogenes were more abundant in *X. fastidiosa* subsp. *pauca* (3%). Fragmented pseudogenes were highest in *X. fastidiosa* subsp. *multiplex* (8%) (Fig. S1A). Among host groups, grapes (92%) consisted of the highest percentage of intergenic pseudogenes, while the “weed/wild” category has the highest proportion of fragmented (11%) pseudogenes and short (16%) pseudogenes. The citrus group had the highest number of long (5%) pseudogenes (Fig. S1B). Considering the country of isolation, strains from Taiwan (89%) had the highest percentage of intergenic pseudogenes. Short (15%) and fragmented (7%) pseudogenes were higher among strains isolated from France. Mexico (5%) had more long pseudogenes (Fig. S1C).

### Blueberry host-specific genes in *X. fastidiosa*

#### Genes intact in strains isolated from blueberries but pseudogenized in strains isolated from other hosts

To identify possible sequences that may be involved in *X. fastidiosa* host specificity for blueberry, we first identified pseudogenes in strains from other hosts that were intact (presumably functional) in blueberry strains, with the hypothesis that strains infecting other hosts were discarding genes that may be useful for blueberry infection. To narrow down this list, we selected only pseudogene sequences that were shared among strains infecting a particular host (Table 2). We detected shared pseudogene sequences within the citrus, coffee, almond, mulberry, buckthorn, plum, and olive groups. In contrast, no common pseudogene sequences were found within the grape group. Notably, the mulberry group exhibited the highest number of shared pseudogene sequences with 107 matches, while the plum group had the fewest hits, totaling only 5.

**TABLE 2:** Shared pseudogene sequences among different strains isolated from the same host

Host group^*a*^	No. of shared pseudogene sequences^*b*^	Percentage of shared sequences^*b*^
Citrus	97	7.6
Mulberry	107	11.6
Olive	9	0.52
Coffee	10	0.66
Almond	7	0.26
Grapes	0	0
Buckthorn	10	2.12
Plum	5	0.65

^*a*^ Pseudogene sequences for each strain infecting a particular host were selected with the criteria for having its corresponding intact gene present in all blueberry strains.

^*b*^ Pseudogene sequences for each strain infecting a particular group were compared among each other, and those that were shared among all strains from the same host were considered here. CRB-BLAST was used to identify shared sequences by sequence similarity with a threshold of ≥90%.

To further narrow down the pseudogene list, we considered every possible pair of host groups and identified the pseudogenes shared between these pairs (using CRB-BLAST with a sequence identity of ≥90%) (Fig. S2). The largest number of shared pseudogene sequences occurred between the mulberry and citrus groups, totaling 16, while the coffee–plum, olive–plum, and coffee–buckthorn pairs each displayed only one shared sequence. Considering this final list of pseudogenes, we found that three sequences were shared in at least three host pairs (Table 3). Interestingly, the sequence SeqA was shared among 14 host pair combinations, SeqB was found in six pairs, while SeqC was found in three pairs. All three sequences were missed in the gene calling of AlmaEm3 (coordinates are provided in Table 3); however, when blasted against *X. fastidiosa* genomes, we found intact protein sequences for their corresponding sequences in other *X. fastidiosa* strains.

**TABLE 3:** Pseudogenes and their corresponding intact copies of genes putatively involved in *X. fastidiosa* specificity for blueberries

Pseudogene sequence	Pseudogene length (nt)	Intact gene in AlmaEm3 (coordinates^*a*^)	Genomic context^*b*^	Intact copy length (nt)	Intact copy protein loci^*c*^ (strain hit)	Intact copy annotation^*c*^
Shared in at least three host-pair combinations^*d*^						
SeqA	222	1980834–1981313	Different	480	EWG15159 (Mul-MD)	Tyrosine-type recombinase/integrase
SeqB^*e*^	146	759888–760034	Same	147	ERI59862 (Griffin-1)	Hypothetical protein
SeqC	260	2302100–2302333	Different	234	ACA13033 (M12)	Hypothetical protein
Shared among strains non-symptomatic in blueberry^*f*^						
Seq1	95	1449238–1454853	Different	5616	QTX27226 (AlmaEm3)	Hemagglutinin
Seq2	143	1503325–1503465	Same	141	AAF85041 (9a5c)	Hypothetical protein
Seq3^*e*^	146	759888–760034	Same	147	ERI59862 (Griffin-1)	Hypothetical protein
Seq4	378	353525–353683	Different	159	AIC11015 (Ann-1)	Hypothetical protein

^*a*^ Coordinates are shown for the whole genome of strain AlmaEm3 available at NCBI.

^*b*^ Genomic context comparison between pseudogene and intact copies. For more information, see text.

^*c*^ Because these sequences were not annotated in the AlmaEm3 genome, we identified the loci and annotation in a different strain.

^*d*^ For the first approach, we selected the sequences that were pseudogenized in strains isolated from other hosts while intact in blueberry-isolated strains (see text for details). The pseudogene sequences listed in this table are found to be pseudogenized in at least three host pair combinations when compared with the intact sequences present in blueberry strains.

^*e*^ SeqB and Seq3 sequences are similar and were found independently using both approaches.

^*f*^ Four pseudogene sequences shared by *X. fastidiosa* strains that do not cause symptoms in blueberries. These were selected based on previous research (see text) by comparison of pseudogene sequences shared among non-symptomatic strains against symptomatic strains tested on blueberries.

#### Genes pseudogenized in strains asymptomatic in blueberry

In addition to the approach described above, we conducted an additional analysis to find blueberry host-specific genes. Based on previous experiments from our laboratory conducted in the greenhouse, we compiled a list of strains symptomatic and asymptomatic in blueberries (14, 19). We used this list to compare pseudogenes among these strains and identify putative host-specific genes. The comparison of pseudogene sequences between asymptomatic and symptomatic strains provided only four sequences that were pseudogenized in strains causing no symptoms on blueberry plants but remained intact in strains that have been isolated and cause symptoms on blueberry plants (Table 3). Again, all these sequences were missed in gene calling in AlmaEm3 (except Seq1), therefore we identified the intact copies in different strains. Seq3 was the same as SeqB as noted in Table 3.

## DISCUSSION

**Fig 4:**
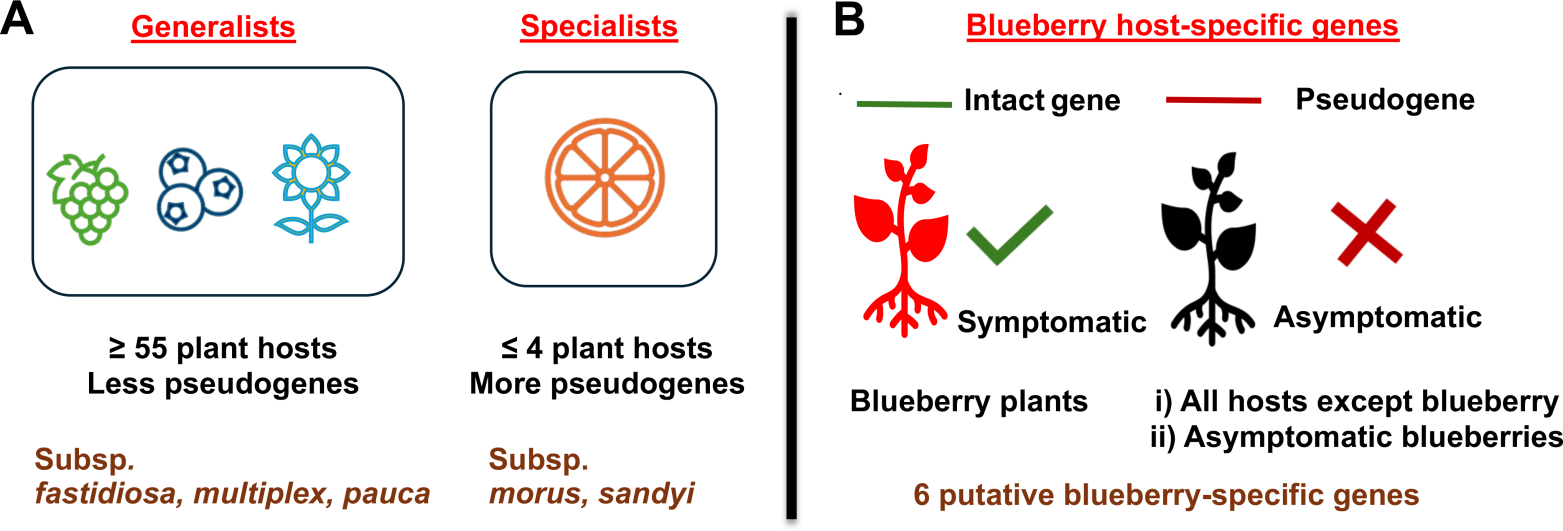
Diagram summarizing the main findings of this study. (**A**) Based on the percentage of pseudogenes on different *X. fastidiosa* strains, subspecies were classified into generalist or specialist categories. This could be used as a tool to assess the risk of certain strains infecting a higher number of hosts. (**B**) Using information on the host of isolation (i) and assessment of symptoms (ii) caused by certain strains in blueberry, we compared intact vs pseudogenes content among a group of strains. When using these two approaches, six genes were identified as putatively important for specificity to blueberry, including one shared by both analyses.

In this study, we propose that the abundance of pseudogenes in *X. fastidiosa* genomes is an indicator of host range and specialization (Fig. 4A). Bacterial species that have recently developed close relationships with, or dependencies on, eukaryotic hosts are more likely to harbor pseudogenes (49) that can be degraded and eliminated from genomes over time (50), leading to genome reduction. These non-functional segments of DNA can reach high proportions in the genome, and in some rare instances, take up more than half of their size (49), as we observed among *X. fastidiosa* genomes where a few strains had 45%–46% pseudogenization. This suggests that this pathogen is still undergoing substantial genome reduction to adapt to its specialized ecological niche. Identification of pseudogenes is important to understand the physiology, metabolism, and evolutionary adaptation of pathogens and symbionts (37).

Here, we considered that pseudogenized sequences can be a clue for host specificity if the corresponding intact gene sequence is present in strains that cause symptoms in a particular host. Moreover, the high abundance of pseudogenes in a particular genome was considered a sign of host specialization by an ongoing process of genome reduction. On the other hand, a lower content in pseudogenes was considered an indication of broader host range by keeping functions that can be useful for different host colonizations. We modeled these criteria on research performed in human pathogens (51–53). *X. fastidiosa* genomes belonging to five different subspecies used in this study were classified into host generalists and host specialists based on overall pseudogene content and host range. We propose that *X. fastidiosa* subsp. *fastidiosa, multiplex*, and *pauca* might fit in the host generalist category, while subsp. *morus* and *sandyi* could be classified as host specialists. This correlates with the information from a database of naturally infected host plant species published by EFSA 2023 (54), where *X. fastidiosa* subsp. *multiplex, fastidiosa,* and *pauca* have wider reported natural host range (213, 58, and 55 host plant species, respectively) as compared to *X. fastidiosa* subsp. *sandyi* (seven hosts) and *morus* (four hosts). Noteworthy is that while *X. fastidiosa* subsp. *fastidiosa* had the lowest number of pseudogenes (18.18%), and *X. fastidiosa* subsp. *pauca* had a higher one (21.36%), both have a similar number of host plant species described so far. If our classification is correct, we predict that more hosts could be found for *X. fastidiosa* subsp. *fastidiosa* in the future. However, subsp. *pauca* seems to be intermediate between generalists and specialists according to their pseudogene numbers, so it may be that the number of hosts for this subsp. will not dramatically increase in the future.

The primary limitation of this study was the unbalanced representation of genomes across categories, i.e., subspecies, host, and country of isolation. Despite this limitation, we were still able to establish a relationship between pseudogenes and host generalist/specialist isolates. A relatively higher percentage of pseudogenes were identified for every strain of *X. fastidiosa* with Pseudofinder compared to Prokaryotic Genome Annotation Pipeline (PGAP) on NCBI (28). However, the differences observed in pseudogene predictions among PGAP and Pseudofinder highlight the complexity of pseudogene identification (37). Although the *X. fastidiosa* genomes used in this study were complete, still, the quality of the provided genomes could have a significant impact on all these pseudogene calls (37).

We took advantage of the availability of whole transcriptome data to further explore and cross-examine the classification of pseudogenes by Pseudofinder. Unfortunately, RNA-Seq data were available just for the strain Temecula1 (38), limiting our availability of comparing different transcribed sequences with our pseudogene analysis database. We confirmed that the great majority of the sequences classified as pseudogenes for this strain by Pseudofinder were not transcribed. The only exception was nine sequences that were transcribed *in vitro* (38) and considered as pseudogenes with our classification. This could be explained by technical reasons because the transcriptome analyses data might be biased by the conditions in which experiments were performed *in vitro*. Another explanation is a misclassification of these sequences by Pseudofinder. Interestingly, eight of these sequences landed in intergenic regions, except one sequence, which was predicted to be fragmented, so this may be an indication that these sequences may have regulatory functions. Noteworthy is that although pseudogenes have been considered to be useless for the organism function, in recent years, it has been shown in eukaryotes that in some cases, their transcripts can be processed into short interfering RNA that can regulate important functions (55). This will have to be tested experimentally for bacteria in the future.

Among the types of pseudogenes—truncated/short, long, fragmented, and intergenic—a comparison was made considering three groups, i.e., subspecies, host groups, and countries. In all three groups, the percentage of intergenic pseudogenes was higher, followed by short pseudogenes, fragmented pseudogenes, and long pseudogenes. We are not aware of any studies understanding the evolutionary implications of the different types of pseudogenes.

Although we did not find much variability in pseudogene content among different *X. fastidiosa* genomes, surprisingly, isolates from weed/wild hosts showed almost half of their genomes pseudogenized, suggesting a high host specificity to these plant species. This may be partly explained by the fact that in highly specialized niches, certain genes, including those related to metabolism or defense, become inactivated when their functions are no longer essential (56, 57). Data from natural infection of wild species with *X. fastidiosa* and mechanical inoculation studies in greenhouse suggest that infection with *X. fastidiosa* is usually asymptomatic; therefore, it is very difficult to assess the possible host range of *X. fastidiosa* (14, 58). Asymptomatic hosts play a crucial role in disease epidemiology because they can act as natural reservoirs for the pathogen and serve as sources of infection for cultivated crops (59). Because wild and weed hosts are not under agricultural production management, the association of the pathogen and host can be extended for longer times. We speculate that this can lead to long-lasting associations leading to pathogen specialization. Unfortunately, *X. fastidiosa* strains from weed/wild plant species in our database belonged to only *X. fastidiosa* subsp. *fastidiosa* and subsp. *multiplex*, which was a limitation of our analysis. In contrast, the lowest proportion of pseudogenes found in strains isolated from grapevine and almonds could be an indication of their ability to infect multiple host species. Cross-inoculation studies of strains isolated from grapes and almonds highlight the ability of grape strains to infect almonds, while almond strains did not produce typical symptoms in grapes. Moreover, in another study, almond strains were able to infect multiple host species (13, 18, 60). Similarly, grape strains successfully colonized blueberry plants (14). Furthermore, reciprocal inoculation studies of *X. fastidiosa* subsp. *pauca* strains in citrus and coffee (61, 62) suggest that some strains of this subspecies may exhibit host specificity, possibly linked to its higher pseudogene content. However, our knowledge about host range studies is still very limited, and the relationship between different isolates and host specificity has not yet been clearly established.

The highest number of genomes in our collection was from strains that were isolated in the United States, with the majority belonging to *X. fastidiosa* subsp. *fastidiosa.* Interestingly, strains isolated from grapes and from the United States had low numbers of pseudogenes, probably indicating a long pathogen–host relationship (Pierce’s disease of grapevine was first observed in the United States in the 1880s). Similarly, strains from Costa Rica in Central America, a region that has been proposed to be the center of origin of *X. fastidiosa* subsp. *fastidiosa* (63), had a lower number of pseudogenes. Therefore, it can be hypothesized that the regions/countries where *X. fastidiosa* have been established for longer times have more potential to infect a wide range of hosts. In contrast, the isolation of European strains is more recent, where the first *X. fastidiosa* infection was identified in 2013 in Apulia (64). European strains contained a higher proportion of pseudogenes, suggesting that these strains are still undergoing evolutionary adaptation to their environments, likely due to relatively recent infections in these regions (7). *A priori* strains from Brazil were expected to have a lower content of pseudogenes because it is predicted that *X. fastidiosa* in that country has been present for a few centuries (65). Nevertheless, they had a higher content of pseudogenes. It is important to remember that all these discussions are speculations based on our studies with a less-than-ideal database.

Our idea of comparing the intact sequences of strains having blueberry as a potential host plant, with the pseudogene sequences of strains isolated from other host plants, was informative in terms of finding the factors or genes that are still intact/functional in blueberry strains and may play a role in causing symptoms on these plants (Fig. 4B). We first identified pseudogenes shared by strains that were isolated from hosts other than blueberry. Interestingly, we could not find any pseudogenized sequences (and intact in blueberry strains) common among all grape strains, which might be attributed to geographical isolation of strains infecting grapes and blueberries, and possible sharing of these two hosts by some strains. Although all the grape strains in our database belong to *X. fastidiosa* subsp. *fastidiosa* and all blueberry strains are *X. fastidiosa* subsp. *multiplex* strains, recent studies have shown that *X. fastidiosa* subsp. *fastidiosa* can infect blueberries (14, 19, 23) and *X. fastidiosa* subsp. *multiplex* can infect grapes (66).

By comparing the genomes of strains known to infect or not infect blueberries, we were able to identify possible host-specific sequences. The approach of finding common pseudogenes among strains belonging to each host group was helpful to narrow down the list of sequences putatively involved in blueberry infection. Interestingly, one sequence annotated as a hypothetical protein was common among the two different approaches and should be further studied. Future analysis should confirm that these sequences are preferentially expressed in strains infecting blueberries, and their function should be assessed experimentally. These sequences could be very important in elucidating the factors responsible for host specificity in different strains of *X. fastidiosa*.

In summary, our study proposes a possible relationship between pseudogene abundance and host specificity in *X. fastidiosa*, one of the most economically significant plant pathogens in agricultural production. The differentiation between host specialist and generalist strains based on pseudogene content could be crucial to enhance the understanding of this pathogen behavior. Host generalist strains, which can infect a wider range of plant species, pose a greater challenge in disease control, whereas host-specific strains, limited to a few hosts, are comparatively easier to predict in terms of their spread. Moreover, the identified pseudogenes from our study may offer valuable insights into the factors governing host specificity, particularly in key crops like blueberries. This foundational knowledge not only advances our understanding of *X. fastidiosa* but also lays the groundwork for future translational studies aimed at improving crop surveillance and protection strategies.

## Data Availability

All raw data and scripts used in this study are available on the following GitHub site: https://github.com/dlf-xyl/Pseudogenes.

## SUPPLEMENTAL MATERIAL

Fig. S1 aem.02070-24-s0001.tif Percentage of pseudogenes as classified by Pseudofinder into types in different strains of *X. fastidiosa*.

Fig. S2 aem.02070-24-s0002.tif Number of shared pseudogene sequences between all chosen host group pairs.

Supplemental legends aem.02070-24-s0003.docx Legends for Fig. S1 and S2.

Table S1 aem.02070-24-s0004.xlsx Whole genome sequences used in this study.

ASM does not own the copyrights to Supplemental Material that may be linked to, or accessed through, an article. The authors have granted ASM a non-exclusive, world-wide license to publish the Supplemental Material files. Please contact the corresponding author directly for reuse.
