# Erratum for Kaur et al., “Pseudogenes and host specialization in the emergent bacterial plant pathogen *Xylella fastidiosa*”

**DOI:** 10.1128/aem.01781-25

**Published:** 2025-09-23

**Authors:** Navdeep Kaur, Neha Potnis, Leonardo De La Fuente

## ERRATUM

Volume 91, no. 5, e02070-24, 2025, https://doi.org/10.1128/aem.02070-24. Page 5, line 37: “strain XF26_VALVAL072_TX (isolated from Ragweed, *Palmer amaranthus*)” should read “strain XF26_VALVAL072_TX (isolated from giant ragweed, *Ambrosia trifida* var. *texana*).”

