# Supplementary figures and images for: Pseudogenes and host specialization in the emergent bacterial plant pathogen Xylella fastidiosa
Source: Appl Environ Microbiol. 2025 Apr 10;91(5):e02070-24. doi: 10.1128/aem.02070-24 (PMC12093968; doi:10.1128/aem.02070-24)

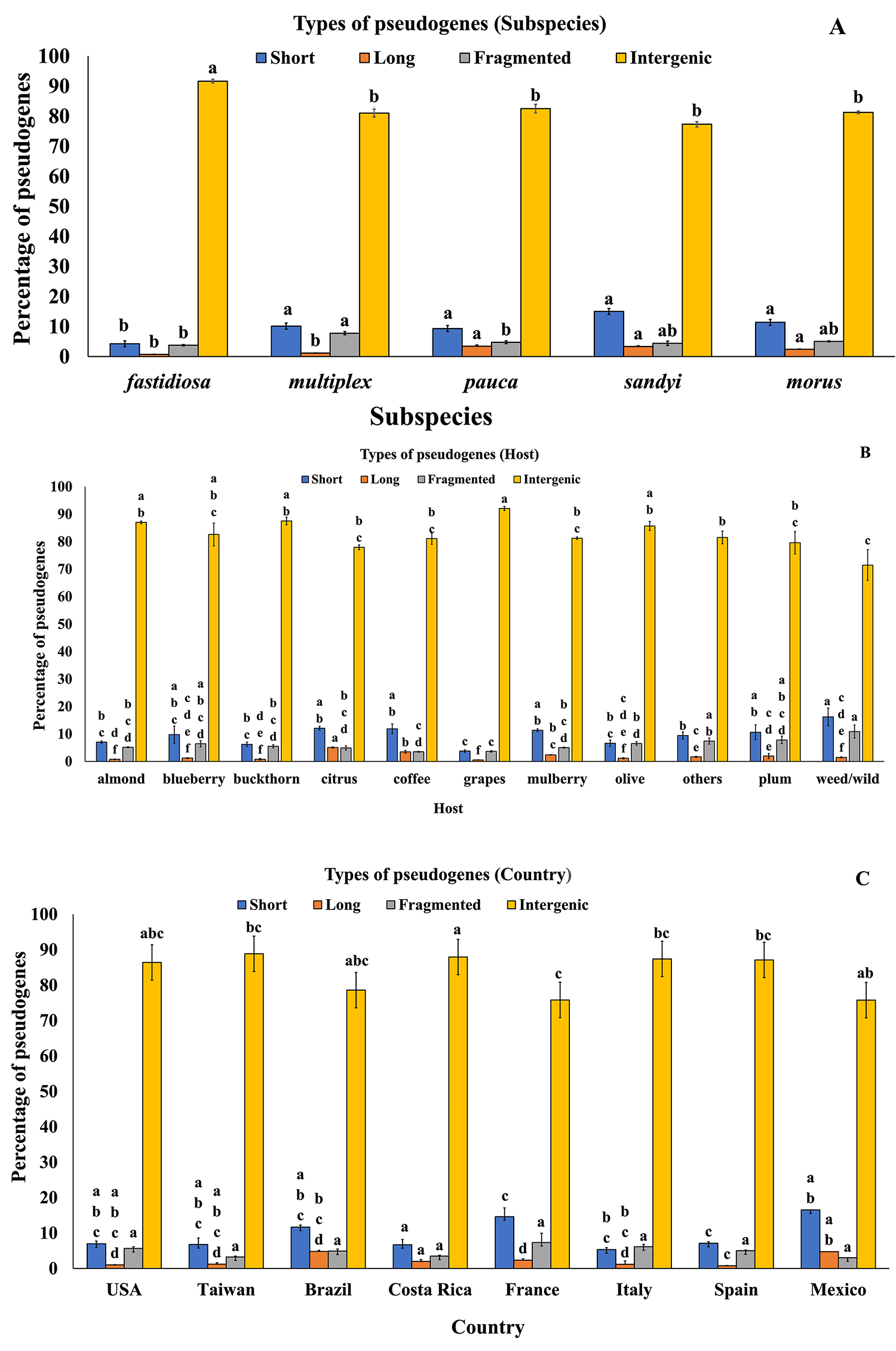

Supplement: Fig. S1 — Percentage of pseudogenes as classified by Pseudofinder into types in different strains of X. fastidiosa. [file aem.02070-24-s0001.tif]

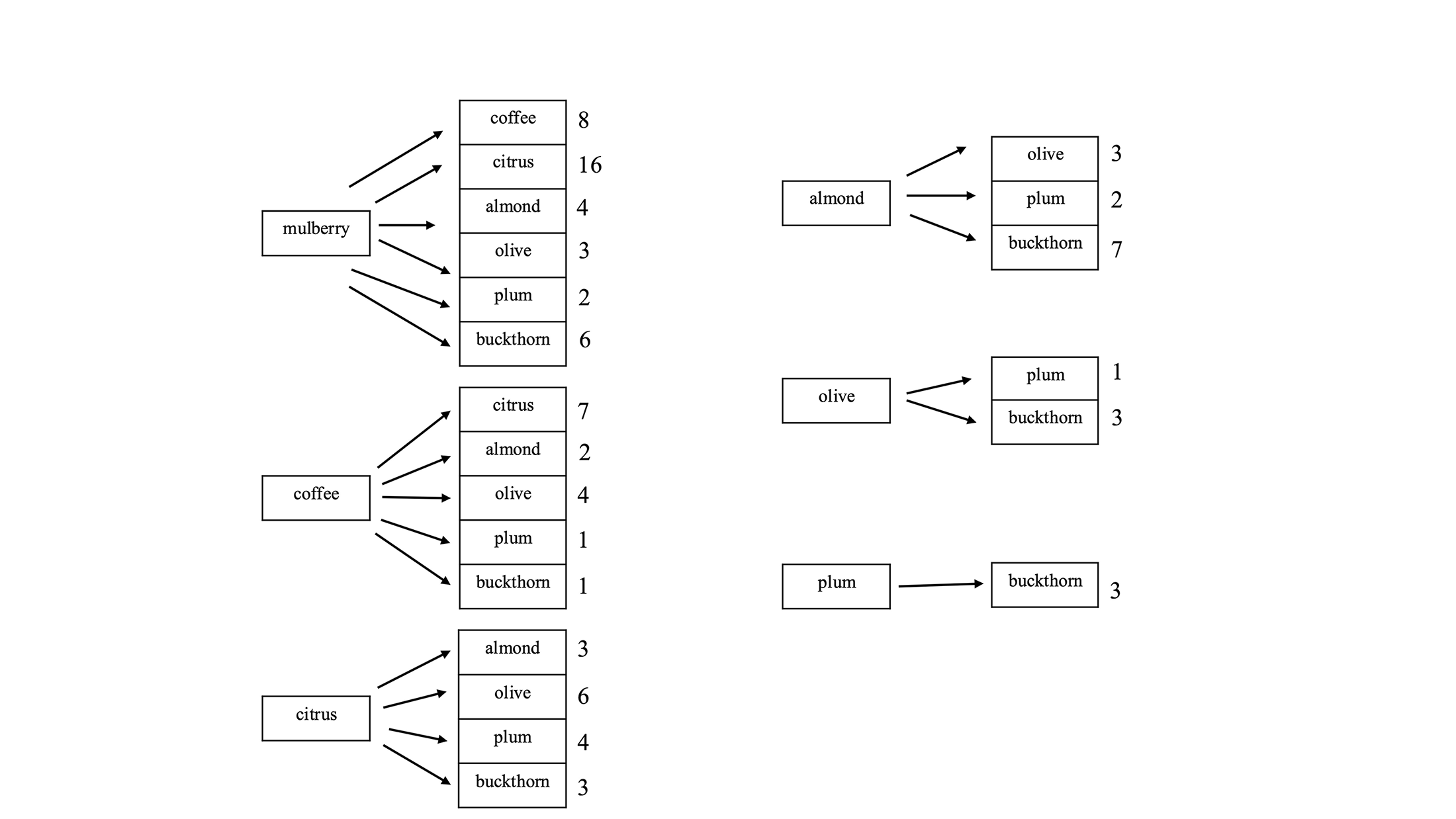

Supplement: Fig. S2 — Number of shared pseudogene sequences between all chosen host group pairs. [file aem.02070-24-s0002.tif]
