# Supplementary material for: Pseudogenes and host specialization in the emergent bacterial plant pathogen Xylella fastidiosa
Source: Appl Environ Microbiol. 2025 Apr 10;91(5):e02070-24. doi: 10.1128/aem.02070-24 (PMC12093968; doi:10.1128/aem.02070-24)
Supplement: Supplemental legends — Legends for Fig. S1 and S2. [file aem.02070-24-s0003.docx]

**Applied and Environmental Microbiology**

**Pseudogenes and host specialization in the emergent bacterial plant pathogen *Xylella fastidiosa***

Kaur, Navdeep^a^, Neha Potnis^a^, and Leonardo De La Fuente^a,*^.

^a^Department of Entomology and Plant Pathology, Auburn University, Auburn, Alabama, USA

*Corresponding author: Leonardo De La Fuente; email: [lzd0005@auburn.edu](mailto:lzd0005@auburn.edu)

**Supplemental Materials**

**Supplemental figure legends**

**Fig. S1**. Percentage of pseudogenes as classified by Pseudofinder into types in different strains of *X. fastidiosa*. Data on short, long, fragmented and intergenic pseudogenes were grouped according to subspecies classification (A), host (B), and country of isolation (C).

**Fig. S2**. Number of shared pseudogene sequences between all chosen host group pairs. Scheme summarizing the comparisons of pseudogenes and intact genes between strains isolated from different hosts. These comparisons were made to narrow down the list and identify genes that were pseudogenized in strains isolated from hosts other than blueberries, while strains isolated from blueberry had intact copies of them. The goal was to identify putative blueberry-specific genes (see Table 3).
